# Neuronal nicotinic acetylcholine receptor antibodies in autoimmune central nervous system disorders

**DOI:** 10.3389/fimmu.2024.1388998

**Published:** 2024-05-28

**Authors:** Maria Pechlivanidou, Aigli G. Vakrakou, Katerina Karagiorgou, Erdem Tüzün, Eleni Karachaliou, Elisabeth Chroni, Theodora Afrantou, Nikolaos Grigoriadis, Christina Argyropoulou, Nikolaos Paschalidis, Elif Şanlı, Aikaterini Tsantila, Maria Dandoulaki, Elpinickie I. Ninou, Paraskevi Zisimopoulou, Renato Mantegazza, Francesca Andreetta, Leon Dudeck, Johann Steiner, Jon Martin Lindstrom, Dimitrios Tzanetakos, Konstantinos Voumvourakis, Sotirios Giannopoulos, Georgios Tsivgoulis, Socrates J. Tzartos, John Tzartos

**Affiliations:** ^1^ Tzartos NeuroDiagnostics, Athens, Greece; ^2^ First Department of Neurology, School of Medicine, Aeginition Hospital, National and Kapodistrian University of Athens, Athens, Greece; ^3^ Department of Biochemistry and Biotechnology, University of Thessaly, Larissa, Greece; ^4^ Department of Neuroscience, Aziz Sancar Institute for Experimental Medical Research, Istanbul University, Istanbul, Türkiye; ^5^ Second Department of Neurology, School of Medicine, “Attikon” University Hospital, National and Kapodistrian University of Athens, Athens, Greece; ^6^ Department of Neurology, School of Medicine, University of Patras, Patras, Greece; ^7^ Second Department of Neurology, “AHEPA“ University Hospital, Aristotle University of Thessaloniki, Thessaloniki, Greece; ^8^ Department of Neurology, Nicosia General Hospital, Nicosia, Cyprus; ^9^ Mass Cytometry-CyTOF Laboratory, Center for Clinical Research, Experimental Surgery and Translational Research, Biomedical Research Foundation of the Academy of Athens (BRFAA), Athens, Greece; ^10^ Department of Neurobiology, Hellenic Pasteur Institute, Athens, Greece; ^11^ Neuroimmunology and Neuromuscular Diseases Unit, Fondazione I.R.C.C.S. Istituto Neurologico Carlo Besta, Milan, Italy; ^12^ Department of Psychiatry and Psychotherapy, Otto-von-Guericke-University Magdeburg, Magdeburg, Germany; ^13^ Department of Neuroscience, Medical School, University of Pennsylvania, Philadelphia, PA, United States; ^14^ Department of Pharmacy, University of Patras, Patras, Greece

**Keywords:** neuronal nicotinic acetylcholine receptor, nAChR subunit α4, autoimmunity, autoimmune encephalitis syndromes, autoimmunity-driven cognitive impairment

## Abstract

**Background:**

Neuronal nicotinic acetylcholine receptors (nAChRs) are abundant in the central nervous system (CNS), playing critical roles in brain function. Antigenicity of nAChRs has been well demonstrated with antibodies to ganglionic AChR subtypes (i.e., subunit α3 of α3β4-nAChR) and muscle AChR autoantibodies, thus making nAChRs candidate autoantigens in autoimmune CNS disorders. Antibodies to several membrane receptors, like NMDAR, have been identified in autoimmune encephalitis syndromes (AES), but many AES patients have yet to be unidentified for autoantibodies. This study aimed to develop of a cell-based assay (CBA) that selectively detects potentially pathogenic antibodies to subunits of the major nAChR subtypes (α4β2- and α7-nAChRs) and its use for the identification of such antibodies in “orphan” AES cases.

**Methods:**

The study involved screening of sera derived from 1752 patients from Greece, Turkey and Italy, who requested testing for AES-associated antibodies, and from 1203 “control” patients with other neuropsychiatric diseases, from the same countries or from Germany. A sensitive live-CBA with α4β2-or α7-nAChR–transfected cells was developed to detect antibodies against extracellular domains of nAChR major subunits. Flow cytometry (FACS) was performed to confirm the CBA findings and indirect immunohistochemistry (IHC) to investigate serum autoantibodies’ binding to rat brain tissue.

**Results:**

Three patients were found to be positive for serum antibodies against nAChR α4 subunit by CBA and the presence of the specific antibodies was quantitatively confirmed by FACS. We detected specific binding of patient‐derived serum anti‐nAChR α4 subunit antibodies to rat cerebellum and hippocampus tissue. No serum antibodies bound to the α7-nAChR-transfected or control-transfected cells, and no control serum antibodies bound to the transfected cells. All patients positive for serum anti‐nAChRs α4 subunit antibodies were negative for other AES-associated antibodies. All three of the anti‐nAChR α4 subunit serum antibody-positive patients fall into the AES spectrum, with one having Rasmussen encephalitis, another autoimmune meningoencephalomyelitis and another being diagnosed with possible autoimmune encephalitis.

**Conclusion:**

This study lends credence to the hypothesis that the major nAChR subunits are autoimmune targets in some cases of AES and establishes a sensitive live-CBA for the identification of such patients.

## 1 Introduction

The nicotinic acetylcholine receptors (nAChRs) belong to the superfamily of homologous “cys-loop” ligand-gated ion channels formed by five homologous subunits. Neuronal nAChRs include several subtypes whose subunits are selected from 12 different subunits (α2-α10, β2-β4) (1–5). Neuronal nAChRs are found throughout the central (CNS) and peripheral nervous system (PNS) (6, 7) and mediate essential physiological processes, such as arousal, learning and memory, reward, and motor control (1, 2). The heteromeric α4β2 and the homomeric α7-nAChR subtypes have been described as the most abundant in the CNS, whereas α3β4 is the most common nAChR subtype located at the peripheral ganglia (8).

Autoimmunity to neuronal nAChRs is uncommon, except for autoantibodies to the α3 subunit containing nAChR (α3β4-nAChR), which are detected and are likely pathogenic in patients with Autoimmune Autonomic Ganglionopathy (AAG) (9, 10). Limited evidence supports the presence of antibodies to the α3 subunit of nAChRs in some patients with Autoimmune Encephalitis who also have dysautonomia (11, 12). Except for autoantibodies against nAChRs-α3-subunit, autoantibodies against other subtypes of neuronal nAChRs have not received much research interest and appear to be exceedingly uncommon in the population. Very rarely, α7-nAChR antibodies have been reported in Rasmussen’s encephalitis, schizophrenia (13) and in some myasthenia gravis patients (14–17). Regarding α4β2- and α7-nAChR autoimmunity, there is one study showing the presence of such antibodies in a patient with AAG and late-onset encephalopathy (18) and non-encephalopathic seropositive AAG patients have been identified to possess antibodies against the α4 subunit of nAChRs (19, 20).

Autoimmune encephalitis syndromes (AES) constitute a rapidly growing disease spectrum characterized by antibody-mediated neuronal or synaptic dysfunction, resulting in limbic encephalitis, NMDAR encephalitis, brainstem encephalitis, cerebellitis, or encephalomyelitis. However, many autoimmune encephalitis patients have no detectable antibodies using current techniques. The discovery of new antibodies is important for clinical diagnosis, allows the prognostic prediction, precision immunotherapy, and early immunotherapy, all of which enhance the final outcome of affected patients (21, 22). The main presenting clinical manifestations of the AES spectrum involve altered mental status, behavior or personality change, cognitive decline and epilepsy. Many epilepsy genes encode ion channels or other proteins involved in neuronal excitability and synaptic transmission. The first gene linked to idiopathic frontal lobe epilepsy was CHRNA4, which encodes the α4 subunit of the α4β2-nAChR (4, 23) and data from mutant α4-containing nAChRs expressed in Xenopus oocytes revealed that such dominant mutations on the M2 domain might cause alterations in the channel properties of nAChRs, increasing the susceptibility to epilepsy. Studies including either the α4 subunit-nAChR knock out mice causing dysfunctional nAChRs or reduced numbers of functional α4β2-nAChRs, have demonstrated the essential role of these neuronal receptors in complex physiological functions and sophisticated brain circuits i.e., reduced locomotor activity (24). Thus, we investigated the presence of α4β2- and α7-nAChR antibodies in several autoimmune CNS disorders and postulated that they could have pathogenetic relevance.

In the present study, we aimed to develop a live CBA that selectively detects the potentially pathogenic antibodies targeting the major neuronal nAChR subtypes (α4β2- and α7-nAChR), based on previous knowledge derived from our CBA for antibodies to ganglionic nAChR α3 subunit (9), and assessed its use for the identification of such neuronal autoantibodies in previously seronegative cases of autoimmune CNS disorders belonging to the AES spectrum.

## 2 Materials and methods

### 2.1 Standard protocol approvals, registrations and patient consents

This study was performed according to the Declaration of Helsinki and was approved by the Institutional Review Boards of the Attikon University Hospital (protocol code B’ NEUR. EBD 280/17.5.21; date of approval: 27 July 2021) and followed local institutional review board guidelines. Written informed consent was obtained from all the subjects involved in the study.

### 2.2 Subjects

We collected and screened sera derived from 1752 patients who requested testing for AES-associated antibodies from Athens, Turkey and Milan and 1203 ‘‘control patients’’ suspected for other neuropsychiatric diseases including NMO, myositis, epilepsy, Creutzfeldt-Jacob disease, Schizophrenia, Major depression and Alzheimer’s disease, from the aforementioned countries and Germany.

### 2.3 Cell culture and transfection

HEK293 cells were maintained in Dulbecco’s modified Eagle’s medium (DMEM) containing 10% fetal bovine serum (FBS) and 1% penicillin-streptomycin at 37°C in 5% CO2. Various parameters were tested in preliminary experiments to identify the best conditions. The final selected conditions were as follows: cells were seeded on culture dishes and transiently transfected with a mixture of plasmids pCMV6-XL5-CHRNA4 or pCMV6-XL4-CHRNA3, pCMV6-XL5-CHRNB2, pCMV6- XL5-TMEM35 (NACHO), and pCMV6-XL5-RIC3 (OriGene, Herford, Germany); 3.7 μg/plasmid for α4β2 or α3β2, 5 μg/plasmid for α7-nAChR 3.4 μg for NACHO and 4 μg RIC3 per 100 mm culture dish in principle as described (9); or 4 μg control vector AQP4-myc, using jet- PRIME kit transfection reagent (Polyplus jetPRIME, France). Cells were treated with 1 mM nicotine (N3876, Sigma), or 5 mM DL-α-Difluoromethylornithine hydrochloride hydrate (DFMO) (25) (1003440802, Sigma), for 24 hours before analysis. Cells were washed to remove the ligand nicotine or DFMO before incubation with the test sera.

### 2.4 Cell-based assay

All sera were screened using the ‘‘live’’ CBA with HEK293 cells expressing the major neuronal nAChR subtypes (α4β2 and α7-nAChR). Forty-eight hours post-transfection, cells were washed with DMEM/0.46% w/v N-(2-hydroxyethyl)-piperazine-N’-(2-ethanesulfonic acid) (HEPES) buffer (DMEM-HEPES) in principle as described (9). CBA involved incubation of transfected cells with serum (1/10 dilution in 1% bovine serum albumin in DMEM-HEPES buffer). After 1 hour, cells were washed 3 times with DMEM-HEPES buffer and fixed immediately with 4% paraformaldehyde (PFA) for 10 minutes. Fixed cells were incubated with rabbit anti-Human IgG (Invitrogen) at 1/750 dilution for 1 hour, followed by incubation with goat anti-Rabbit IgG Alexa Fluor-568 (Invitrogen), as the third antibody, at 1/750 dilution for 1 hour (all at RT). The use of the third antibody increased the signal, without increasing the background, and therefore, it increased the sensitivity (data not shown). Microscopy was performed under blinded conditions by 2 or 3 independent observers. The Olympus microscope CKX-41 was used, and images were analyzed using Infinity Analyze-6.5 Lumenera software. As negative controls and to evaluate the specificity of the transfection process, AQP4-transfected HEK293 cells expressing AQP4 as a different membrane protein, were used. Sera were subsequently tested at serial dilutions to determine assay’s sensitivity, and results were expressed as the highest positive dilution. All positive sera were also tested using HEK293 cells transfected with α3β2- and α7-nAChRs.

The colocalization of the binding of positive serum antibodies with the rat anti-α4 subunit mAb299 or anti-β2 subunit mAb295 monoclonal antibodies (1:20, hybridoma cell supernatant (26–30)) was tested by their co-incubation with α4β2- nAChR–expressing cells, followed by simultaneous incubation with mouse anti-Human IgG Alexa Fluor-488 (Invitrogen) and goat anti-Rat IgG Alexa Fluor-568 (Invitrogen) at 1/750 dilution for 1 hour. Cell incubation with only mAb or serum showed no or minimal background binding (data not shown). For the specific detection of the α7 subunit, transfected HEK293 cells expressing α7-nAChR were incubated with fluorescent α-bungarotoxin (**Supplementary Figure 1**).

### 2.5 Indirect immunohistochemistry

Frozen 10-μm-thick sections of rat brain were fixed in PFA overnight (O/N), blocked at RT with blocking solution and incubated with patients and control sera (1:50) at 4 ◦C O/N. The following day, brain sections underwent washes and were incubated for 2 h with secondary biotinylated anti-Human IgG (1:1,000, BA-3000, Vector Laboratories, Newark, CA, USA) at RT. Sections were then counterstained with hematoxylin and mounted with Mounting Medium. Stained histological sections were photographed with an inverted fluorescence microscope (Leica CTR6000) using a digital camera at 5X magnification and the LAS software (4.12).

### 2.6 Fluorescence-activated cell sorting

For each sample, 2 × 10^5^ α4β2-nAChR transfected and untransfected cells were harvested, washed twice with 1mM EDTA in PBS pH 7.4, and incubated for 1 hour at 4°C with patient serum (in blocking buffer 1mM EDTA in PBS-1X plus 10% normal goat serum, 5% BSA). For the experiment, we used two different serum dilutions (1:20, 1:100). Thereafter, the cells were washed twice and incubated with goat anti-Human IgG Alexa Fluor-568 (1:200, Invitrogen) for 30 minutes at 4°C. The cells were washed three times, fixed with 4% PFA for 10 minutes and finally resuspended in 1mM EDTA in PBS, and analyzed by flow cytometry (BD FACSAria™ III Sorter) using the FlowJo software (Version 10, FlowJo Software, 385 Williamson Way Ashland, OR 97520, USA).

## 3 Results

### 3.1 Development of a sensitive CBA for antibodies to α4β2 nAChR subtype

**Figure 1:**
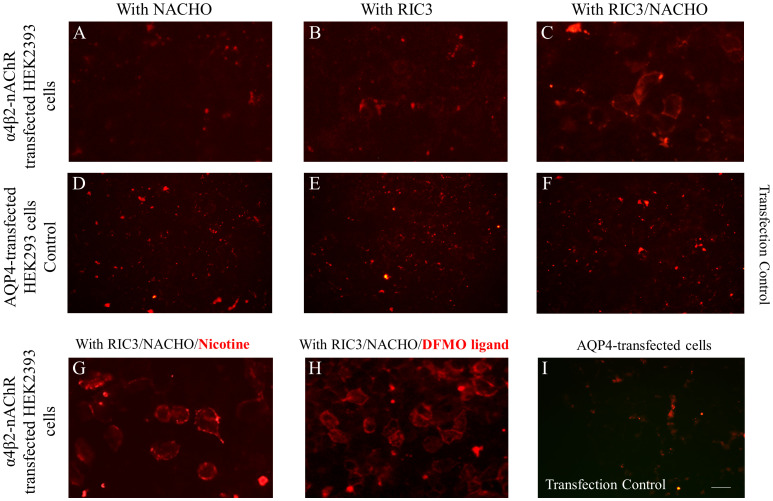
Optimization of the live α4β2-nAChR CBA. All panel images document immunostaining with anti‐nAChR α4 subunit monoclonal antibody (mAb299 at 1/20 dilution) followed by secondary anti-antibody Alexa Fluor 568. HEK293 cells were transfected with plasmids carrying genes encoding α4β2-nAChR subtype and chaperons NACHO **(A)** or RIC3 **(B)** individually, and in combination **(C)** The co-transfection of cells with α4β2-nAChR plasmids and both chaperons RIC and NACHO **(C)** significantly increased the expression of nAChRs which is demonstrated by the cell surface staining, indicative of anti‐α4 subunit antibody binding that correlates with receptor expression. HEK293 cells expressing the transfection control of the experimental conditions **(A-C)**, which is the protein aquaporin-4 (AQP4), did not show any positive signal **(D-F)** as anti‐nAChR α4 subunit antibodies did not bind to the AQP4-expressing cells. Cells co-transfected with α4β2-nAChRs and chaperon plasmids and cultured in the presence of 1mM nicotine **(G)** or 5mM DFMO **(H)** for 24 hours, led to higher α4β2-nAChRs expression. The addition of nicotine or DFMO ligand similarly enhanced the signal compared to the plain medium. In the panel **(I)** with HEK293 cells expressing the transfection control AQP4 of the experimental conditions **(G, H)**, the absence of a positive signal is displayed. Signal in panel **(A, B, D-F, I)** show non‐specific background staining. Scale bar: 20 μm.

We focused our efforts on addressing ways to enhance the surface expression of α4β2- and α7-nAChRs in order to develop more sensitive and accurate live-CBAs. Based on recent literature and our recent efforts (9), we compared the expression of nAChRs co-transfected with NACHO and RIC3 chaperons individually and together and the co-transfection of these two chaperons in parallel with treatment of cell cultures with 1mM nicotine or 5mM DFMO ligands. As shown in **Figure 1** for α4β2-nAChR, the expression of nAChRs was improved by the presence of RIC3 and NACHO chaperones and beyond that achieved, also the incubation in the added presence of either nicotine or DFMO (**Figures 1G, H**) similarly enhanced the expression of nAChR compared to the plain medium, an observation that was achieved under blinded conditions by 3 independent observers. Cells expressing transfection control AQP4 did not show any serum antibody binding with the monoclonal mAb299 anti-nAChR-α4 subunit (**Figures 1D–F, I**). A similar effect was observed for the chaperons and ligands on the expression of α7-nAChRs, monitored by fluorescent α-bungarotoxin (**Supplementary Figure 1**).

### 3.2 Identification of sera positive for antibodies to α4- subunit nAChR by ‘‘live’’ CBA

**Figure 2:**
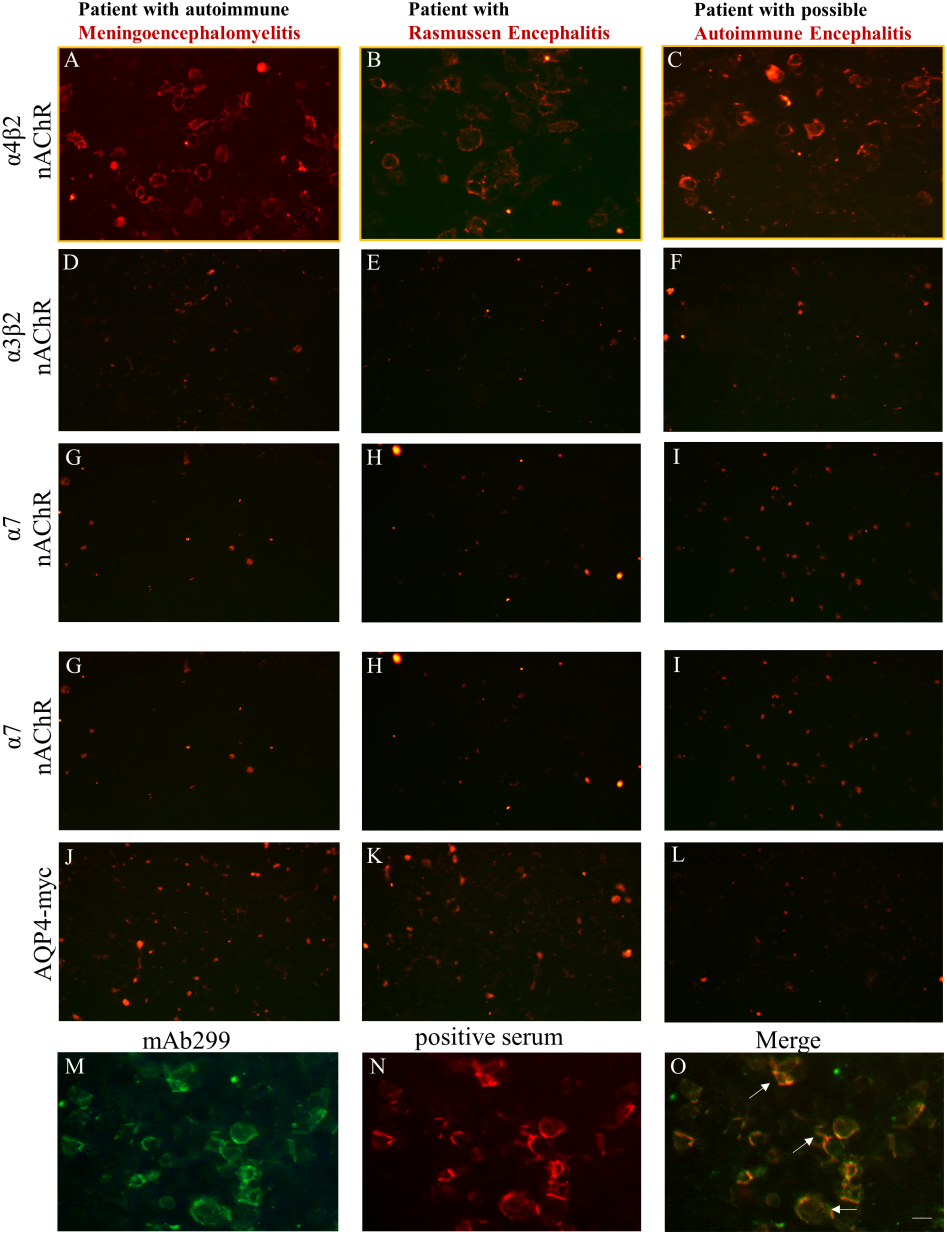
Three patients in the AES spectrum were identified based on the live CBA as being positive for serum antibodies against nAChR α4 subunit epitopes. HEK293 cells expressing α4β2, α3β2, α7-nAChRs, or control AQP4 were incubated with sera derived from patients suspected for AES and found to be positive by the live α4β2-nAChR CBA, followed by secondary and third antibodies. Serum anti-nAChR α4 subunit antibodies were identified in a patient with autoimmune meningoencephalomyelitis **(A)**, in a patient with Rasmussen **(B)**, and in a patient with possible autoimmune encephalitis with movement disorder involving stiffness **(C)**. No staining was observed for these patient serum antibodies when targets were α3β2‐ **(D‐F)** or α7‐nAChRs **(G‐I)** or AQP4 expressed in transfected cells **(J‐L)**. Double immunostaining was performed in α4β2-transfected HEK293 cells using a monoclonal anti-α4 subunit (mAb299) **(M)** and positive sera antibodies to α4 subunit of the α4β2-nAChR derived from patients with AES **(N)**. The overlapping immunoreactivity (white arrows) of the mΑb and the positive serum confirmed the specificity of our α4β2-nAChR CBA **(O)**. Scale bar: 20 μm.

Using the live α4β2-, α7- and α3β2-nAChR CBAs with the optimized conditions, we screened all 1752 patients who requested testing for antibodies associated to AES and we found 3 patients (**Table 1**) positive for serum antibodies against α4β2-nAChR (**Figures 2A–C**). Interestingly, all CBA-positive sera antibodies bound to the α4β2-nAChRs, had antibodies exclusively to the α4 subunit of the α4β2-nAChR subtype and did not have well-established AES-associated neuronal antibodies including anti-NMDAR antibodies. This is further supported by the fact that none of these sera antibodies bound to the α3β2 (**Figures 2D–F**) or α7 nAChRs (**Figures 2G–I**) and control AQP4-transfected cells (**Figures 2J–L**), displaying the specificity of the CBA and of the identified positive serum antibodies. As controls, we screened 1203 “control” patients with other neuropsychiatric diseases and were found to be CBA negative, further indicating a possible association between anti-nAChR α4 subunit antibodies and AES spectrum. In fact, we also screened all test sera for the presence of antibodies to the other major neuronal nAChR subtypes (α3β2‐ and α7‐nAChRs) in addition to the α4β2‐nAChR, based on the study of Yamakawa et al. (11) and to further examine the subunit specificity of the identified anti-nAChR-α4 antibodies. The absence of binding of the three anti-nAChR-α4 antibody-positive sera to α3β2‐ and α7‐nAChRs further confirmed their specificity for the α4 subunit of the α4β2-nAChR.

**Table 1:** Summary of the clinical characteristics of the three α4-nAChR-Abs positive patients.

Patients	Clinical findings	Imaging tests	Electroencephalogram (EEG)	Lumbar puncture findings	Notable Laboratory findings	Treatment
Patient 1	Post-viral headache, vomiting and cervical stiffness, severely decreased bowel motility, urinary retention	T2 weighted MRI: multiple cervical and thoracic fociintramedullary with hyper-intensesignal, PET-CT: increased FDG intake intramedullary indicating inflammation	Normal activity	480 WBC, lymphocytic Type, 35 glucose, increased protein. Type 1 oligoclonal zones (absent)	anti-AQP4 (-), anti-MOG (-), anti-GFAP (-) negative for treponema pallidum, borrelia, West-Nile virus paraneoplastic Abs (-)	IVIg (5 days), IV corticosteroids with recession of symptoms
Patient 2	Drug-resistant, focal motor seizures of the left extremities with retained awareness and gradual establishment of left hemiparesis	T2 MRI: right cerebral hemisphere atrophy, subcortical hyperintensity frontoparietal, left cerebellum atrophy	Continuous recording of slow multiform waves of 1.5-3 Hz with presence of sharp waves at the central-frontoparietal regions of the right hemisphere. Asymmetric background rhythm at the posterior regions, of lower amplitude and frequency on the right hemisphere.	Mild pleocytosis (10 cells)	mGluR3(-), anti-AQP4 (-), anti-MOG (-), anti-GFAP (-)	IVIg (2 days), corticosteroids (3 days), topiramate
Patient 3	altered level of consciousness (psychiatric symptoms, confusion, tardiness), stiffness	No findings in MRI	Focal slow posterior wave activity	No abnormal findings	anti-GAD (-), anti-GFAP (-), anti-AQP4 (-), anti-MOG (-)	Initial response to PLEX, drug resistance (non-responsive to rituximab, mycophenolate mofetil, diazepame, baclofen)

MRI, Magnetic Resonance Imaging; PET-CT, Positron emission tomography/computed tomography; FDG, fluorodeoxyglucose; WBC, white blood cells; AQP4, aquaporin-4; MOG, myelin oligodendrocyte glycoprotein; anti-GAD, anti-glutamic acid decarboxylase; IVIg, Intravenous Immunoglobulin; GFAP, Glial fibrillary acidic protein; mGluR3, metabotropic glutamate receptor-3; PLEX, plasma exchange.

We further performed double immunohistochemistry experiments between patients’ sera and a monoclonal antibody (mAb 299) directed against the α4 subunit of nAChR. The double-labelling experiments revealed an overlapping immunoreactivity (**Figure 2O**) between the anti-α4 subunit mAb299 (**Figure 2M**) and patients’ positive sera antibodies (**Figure 2N**), verifying the specificity and the utility of the α4β2-nAChR live-CBA.

Finally, we validated the findings obtained by the developed live-CBA by conducting FACS analysis, and we confirmed the results by demonstrating that all three patients who were suspected for AES, were found to be positive for antibodies to nAChR α4 subunit (**Supplementary Material**, **Supplementary Figure 2**). These data provide compelling evidence regarding the specificity and sensitivity of the α4β2-nAChR live-CBA.

### 3.3 Binding of human serum -anti-nAChR α4-subunit antibodies to rat brain tissue

**Figure 3:**
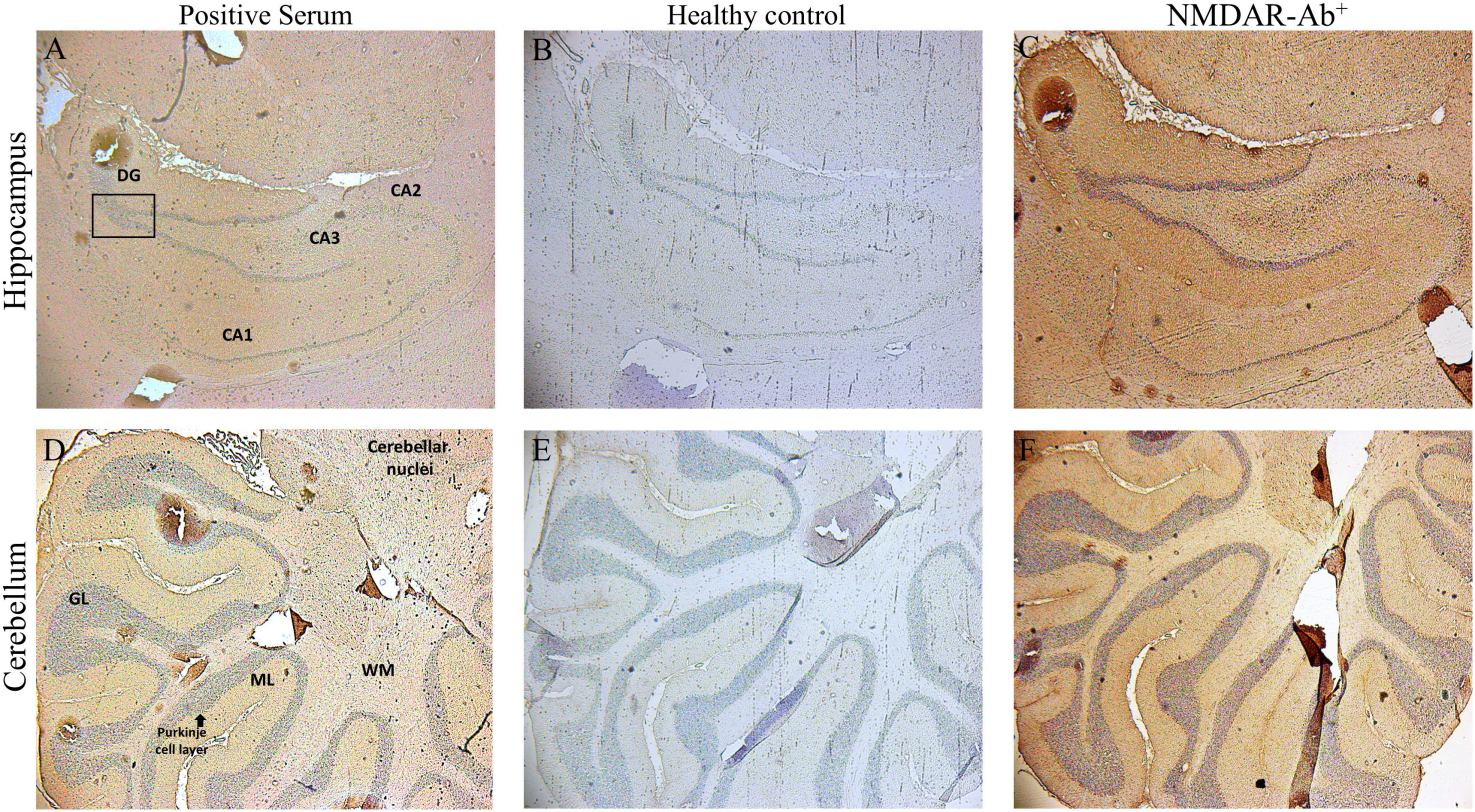
Binding of sera positive for autoantibodies to subunit α4 of the α4β2-nAChRs, to rat brain tissue by Indirect Immunostaining. Sagittal whole rat brain sections were incubated with sera antibodies from the three patients with AES **(A, D)**, from NMDAR encephalitis patients **(C, F)** or from healthy controls **(B, E)**. Representative images are shown the localization of rat α4β2‐nAChRs bound by specific serum antibodies from 1 out of the 3 patients with autoantibodies against subunit α4‐nAChRs in the hippocampus **(A)** and the cerebellum **(D)**, a specific positive signal which was confirmed only by the patients’ sera bearing α4-nAChR-Abs, whereas no specific binding was detected in any region of the rat brain tissue by serum components originated from 1 out of the 10 healthy controls **(B, E)**. Staining and images of rat brain sections with serum antibodies from one NMDAR encephalitis patient as positive control **(C, F)** are depicted. CA1–CA3, hippocampal area cornu ammonis 1–3; DG, dentate gyrus; cerebellar layers. GCl, granular cell layer; ML, molecular layer; WM, white matter.

Finally, we examined whether sera antibodies derived from our three patients recognized neuronal nAChR in rat brain sections. Using indirect immunohistochemistry, all three αpositive sera with anti-nAChR α4-subunit antibodies exhibited a tissue-specific signal with a distinct staining pattern as opposed to the healthy control sera antibodies in tissue sections of the hippocampus and cerebellum regions. Representative images are depicted in **Figure 3**.

### 3.4 Clinical features of three patients with serum anti-nAChR α4-subunit antibodies

The specific clinical characteristics of the three patients with anti-nAChR α4 subunit antibodies fall into the AES spectrum (**Table 1**).

#### 3.4.1 Patient 1

The patient was a 17-year-old male who presented with fever and rash in upper and lower limbs followed by headache, vomiting and cervical stiffness after recovering from mild previous viral infection. During his hospitalization, he developed severely decreased bowel motility and urinary retention. Cerebrospinal fluid (CSF) analysis revealed 480 WBCs/mm^3^, lymphocytic type, 35 mg/100 mL glucose and increased protein 120mg/dl. Brain magnetic resonance imaging (MRI) and electroencephalography (EEG) tests were normal, however, there were multiple cervical and thoracic foci intramedullary with hyperintense signal in T2 weighted MRI with gadolinium enhancement in C3-C4 and in cauda equina, and increased fluorodeoxyglucose (FDG) intake in PET-CT, indicating inflammation. Polymerase chain reaction (PCR) and serology analysis for antibodies to a plethora of viruses and bacteria was negative, including a negative test for West-Nile virus and treponema pallidum, reaching the conclusion that the patient did not have any infectious pathogen during his hospitalization. The patient was also tested negative for MOG, AQP4 antibodies and any other anti-neuronal antibodies associated to AES. The patient was treated with a wide range of antibiotics and was admitted to the intensive care unit (ICU) because of clinical deterioration, involving brainstem involvement and bradycardia. The overall clinical presentation, in addition to the imaging results and the exclusion of other known etiologies, including malignancies, was attributed to possible autoimmune meningoencephalomyelitis as a post-viral event and the patient started therapy with IVIg for 5 days and high doses of corticosteroids. Successful recession of symptoms was observed with this treatment strategy. During the 3-year follow-up, the patient remained asymptomatic, reporting no headache, fatigue, or sleep dysregulation.

#### 3.4.2 Patient 2

The patient was a 15-year-old female who presented with a history of drug-resistant, focal motor seizures of the left extremities with retained awareness since the age of 7 years and gradual onset of muscle weakness of the left upper and lower limbs. Brain MRI revealed findings of right cerebral hemisphere and left cerebellum atrophy and subcortical white matter lesions of high signal on T2 and FLAIR sequences without gadolinium enhancement or calcifications, on the right frontoparietal region. 24h Video–EEG monitoring, revealed focal impaired awareness seizures originating from the right anterior temporal region. Extensive testing for anti-neuronal antibodies and infectious pathogens was performed showing no findings. Specifically, serological tests were negative for VZV, HSV and treponema pallidum. No malignancy was found. The course of the patient’s disease consisted of an initial prodromal stage, which was featured by non-specific, infrequent seizures and mild hemiparesis. The patient then suffered frequent drug-resistant focal motor seizures in the form of EPC that led subsequently to persistent and stable neurological deficits. The above clinical picture along with MRI findings were compatible with the diagnosis of Rasmussen encephalitis. The patient initially received 2 days of IVIg, 3 days of corticosteroids, and multiple regimens of antiepileptic drugs with a moderate response (31).

#### 3.4.3 Patient 3

A 37-year-old female initially presented with rapid-onset altered mental status/decreased level of consciousness, cognitive dysfunction, and psychiatric behavior. Paraclinical tests, lumbar puncture and electromyography (EMG) test, did not reveal any abnormal findings and MRI imaging revealed no structural defects, however, EEG exhibited focal slow waves without epileptic discharges. Extensive testing for anti-neuronal antibodies and infectious pathogens was performed without any findings. After the exclusion of other causes, including malignancy, the patient was treated as a possible autoimmune limbic encephalitis with IVIg and corticosteroids without significant improvement but responded to plasma exchange (PLEX); thus, she was placed on rituximab. Nevertheless, after the induction dose of rituximab, she presented with a new relapse involving episodes of confusion, tardiness, slurred speech, short-term memory impairment, peripheral left arm weakness, paravertebral muscle stiffness and painful muscle spasms. The examination revealed mini-mental state examination (MMSE) of 17 out of 30 with normal cranial nerve testing, normal cerebellar examination and normal deep tendon reflexes. Even while being treated with mycophenolate mofetil, baclofen, and diazepam, the patient suffered a 3^rd^ relapse. Thus, after the exclusion of other causes, we concluded that the most compatible diagnosis was possible autoimmune encephalitis (32).

## 4 Discussion

The main objective of this study was to develop sensitive and specific live-CBAs with cells transfected with the major neuronal nAChRs (α4β2 and α7 subtypes) and to detect antibodies against their extracellular domains in “orphan” AE cases of the AES spectrum. We present novel findings regarding three patients from the 1752 cohort group with suspected AES who were found to be anti-nAChR α4 antibody-positive by both live-CBA and FACS and were also found to be negative for other neuronal and AES-associated antibodies. Our cases presented the clinical phenotypes of meningoencephalomyelitis, Rasmussen encephalitis and possible autoimmune encephalitis. The low frequency of antibodies to α4β2-nAChR we detected in patients suspected for AES was not unexpected since antibodies to several established AES-associated neuronal antigens in patients suspected for AES are similarly infrequent. For example, in Tzartos NeuroDiagnostics, antibodies to AMPAR were detected in 3 out of 1643 suspected patients tested, antibodies to GABA-Α receptor were detected in 1/1040 suspected patients tested and antibodies to GABA-Β receptor were detected in 8/1590 suspected patients tested (data not shown).

The expression of neuronal nAChRs by mammalian cells after induced transfection is usually low, which hinders the development of sensitive CBAs. Therefore, the use of chaperon proteins involved in the biogenesis of nAChRs is essential. NACHO mediates the α7 subunit biogenesis by enhancing protein assembly, protein maturation, trafficking, and the successful insertion of α7 subunit into the cell membrane (33, 34). RIC-3 increased expression of α4 and β2 subunits while having no effect on their assembly into α4β2 receptors (35). We optimized the α4β2- and α7-nAChR expression and the assay sensitivity and specificity based on our CBA for anti-nAChR α3 subunit antibodies (9), by the combined use of the two chaperon proteins and the nicotine or difluoromethylornithine (DFMO) ligand. Nicotine is known to upregulate the expression of nAChRs (36–39). Additionally, a recent publication by Dhara et al. (25) suggested that the ligand DFMO enhances the expression levels of both α7 and α4β2 nAChRs.

Neuronal α4β2- and α7-nAChRs subtypes are abundant in the CNS (2, 4, 40) playing critical roles in brain function, thus making them candidate autoantigens in encephalopathy, AES, and especially in AES-seronegative cases with unidentified autoantibodies. AES is defined as a neurological disease spectrum characterized by the rapid onset of mental confusion, cognitive impairments, seizures and psychiatric symptoms (22, 41). We detected antibodies against the α4-nAChRs in one patient with RE using the live α4β2-nAChR-CBA method. Importantly, the specific patient was found to be CBA negative for antibodies to subunits α7 and α3β2-nAChRs. The first study that revealed the presence of neuronal nAChR antibodies in RE was conducted in 2005 by Watson et al. (42) who screened sera from nine patients with RE and found two patients positive for serum α7-nAChR antibodies. In the present study, we further report two additional cases with the presence of serum anti-nAChR α4 subunit antibodies and the absence of antibodies targeting α7-nAChR. In particular, we identified antibodies targeting the α4 subunit of the α4β2-nAChRs subtype in a patient with autoimmune meningoencephalomyelitis, and another patient diagnosed with possible AES manifesting altered mental status, psychiatric symptoms and confusion. We point on a possible correlation between AES and associated mental and motor symptoms with anti-nAChR α4 subunit antibodies and provide solid evidence with the combination of immunological techniques.

Conversely, Baker et al. traced antibodies against both peripheral and central nAChR subunits (α3, α4 and α7) in the serum of an individual patient displaying AAG clinical phenotype accompanied by late-onset encephalopathy with altered consciousness, nystagmus, ataxia, bladder retention, and symmetric long tract motor signs (18). Interestingly, a Japanese woman with progressive encephalomyelitis with rigidity and myoclonus-like symptoms (PERM) and dysautonomia was found positive for anti-α3-nAChR antibodies (12). Our third case diagnosed with possible AES with movement disorder involving stiffness and found to be positive for anti-nAChR α4 subunit antibodies did not fulfil the criteria for stiff person syndrome Also, Yamakawa et al. reported the detection of antibodies to the ganglionic type α3-AChR using the LIPS assay in the sera of 19 patients with AE syndromes (NMDAR encephalitis, stiff-person syndrome or Isaacs’ syndrome) manifesting autonomic symptoms; 5/19 patients had antibodies to nAChR α3 subunit (11). Τhe authors suggested that the detected ganglionic autoantibodies might be a new molecular entity in the AES-associated immunopathogenesis, however, in our study we did not detect anti-nAChR α3 subunit antibodies in any of the AES cases that we screened. These studies suggest that neuronal nAChR α4 subunit antibodies may be associated with autoimmunity-driven cognitive impairments observed in AES spectrum, however, such cases seem to be rare and their pathological mechanism is yet to be elucidated.

To the best of our knowledge, there are no reports displaying binding and localization of human sera positive for neuronal nAChR antibodies in the animal brain. The IHC analysis demonstrated the binding of the human anti-nAChR α4 subunit antibodies to the CA1 and CA3 hippocampal regions and near dentate gyrus with a synaptic pattern, especially in the hippocampus similar to NMDAR-Αbs. Additionally, we identified a distinct staining pattern in the molecular layer of the rat cerebellar cortex. These findings are both consistent with the regions of abundant expression of α4β2-nAChRs subtypes as reported in the literature (1, 2, 8, 24, 40, 43), and with the MRI findings indicating cerebellar atrophy in one of our three patients. The widespread distribution of nAChRs in several brain along with the specific binding of human sera anti-nAChR α4 subunit antibodies to animal tissue, suggests a possible pathogenic role of these antibodies.

In summary, we show the presence of anti-nAChR α4 subunit antibodies in AES patients. Specifically, three patients positive for anti-nAChR α4 subunit antibodies representing three different AES cases were identified thanks to the novel α4β2-nAChR CBA assay. The pathogenicity of these antibodies is currently unknown, and it remains to be proven whether they are related to an epiphenomenon or possess any functional or destructive pathogenetic effects in CNS tissue. The membrane location of α4β2-nAChR and the absence of an underlying cancer could imply a favorable response to immunotherapy. Our patients showed a heterogeneous response to treatment and only the first patient responded efficiently to IVIG and steroids, whereas the other two had modest or poor responses. Nevertheless, factors like delay in diagnosis and intensity of given immunotherapy could account for treatment outcome. Larger studies are needed to shed light on the treatment outcomes in these patients so that a therapeutic strategy can be established.

One of the limitations of this study is the description of only three seropositive cases with anti-nAChR α4 subunit antibodies. In addition, although all three cases had acute CNS neurological presentations in the absence of an underlying cancer, their phenotypes were variable. Given the rarity of these antibodies, it remains to be seen what the clinical use of this test will be in screening patients with suspected neurological autoimmunity and whether this should be restricted to patients with the above AES syndromes.

## 5 Conclusion

Using a live-CBA we detected serum antibodies against the major nAChR subtype in the CNS (α4β2-nAChR) in three patients with AES. Future studies should focus on the identification of larger patient cohorts of the AES spectrum to evaluate and characterize the clinical phenotype and on the functional characterization of these autoantibodies, which may reveal their role in the pathogenesis of AES. Finally, it remains to be determined whether anti-nAChR α4 subunit antibodies could serve as a biomarker for seronegative cases of the AES spectrum.

## Data availability statement

The original contributions presented in the study are included in the article/**Supplementary Material**. Further inquiries can be directed to the corresponding author.

## Ethics statement

This study was performed according to the Declaration of Helsinki and was approved by the Institutional Review Boards of the Attikon University Hospital (protocol code B’ NEUR. EBD 280/17.5.21; date of approval: 27 July 2021) and followed local institutional review board guidelines. The patients/participants (legal guardian/next of kin) provided written informed consent to participate in this study.

## Author contributions

MP: Writing – review & editing, Investigation, Methodology, Validation, Visualization, Writing – original draft. AV: Investigation, Methodology, Validation, Writing – original draft, Writing – review & editing. KK: Investigation, Methodology, Writing – review & editing. ET: Investigation, Methodology, Resources, Validation, Writing – review & editing. EK: Investigation, Writing – review & editing. EC: Validation, Resources, Writing – review & editing. TA: Resources, Writing – review & editing. NG: Validation, Resources, Writing – review & editing. CA: Resources, Writing – review & editing. NP: Investigation, Methodology, Validation, Writing – review & editing. ES: Investigation, Writing – review & editing. AT: Investigation, Writing – review & editing. MD: Investigation, Writing – review & editing. EN: Investigation, Writing – review & editing. PZ: Writing – review & editing, Investigation. RM: Validation, Funding acquisition, Resources, Writing – review & editing. AF: Resources, Writing – review & editing. LD: Resources, Writing – review & editing. JS: Validation, Funding acquisition, Resources, Writing – review & editing. JL: Resources, Writing – review & editing. DT: Resources, Writing – review & editing. KV: Resources, Writing – review & editing. SG: Resources, Writing – review & editing. GT: Resources, Writing – review & editing, Validation. ST: Funding acquisition, Methodology, Project administration, Validation, Visualization, Writing – review & editing. JT: Conceptualization, Funding acquisition, Methodology, Project administration, Supervision, Validation, Visualization, Writing – original draft, Writing – review & editing.
