# Supplementary material for: Neuronal nicotinic acetylcholine receptor antibodies in autoimmune central nervous system disorders
Source: Front Immunol. 2024 May 28;15:1388998. doi: 10.3389/fimmu.2024.1388998 (PMC11165060; doi:10.3389/fimmu.2024.1388998)
Supplement: Supplementary file 1 [file DataSheet_1.docx]

Supplementary Material

Neuronal Nicotinic Acetylcholine Receptor Antibodies in Autoimmune Central Nervous System Disorders

**Maria Pechlivanidou, Aigli G Vakrakou, Katerina Karagiorgou, Erdem Tüzün, Eleni Karachaliou****, Elisabeth Chroni, Theodora Afrantou, Nikolaos Grigoriadis, Christina Argyropoulou, Nikolaos Paschalidis, Elif Şanlı, Aikaterini Tsantila, Maria Dandoulaki, Elpinickie Ninou, Paraskevi Zisimopoulou, Renato Mantegazza, Andreetta Francesca, Leon Dudeck, Johann Steiner, Jon Lindstrom, Dimitrios Tzanetakos, Konstantinos Voumvourakis, Sotirios Giannopoulos, Georgios Tsivgoulis, Socrates Tzartos and John Tzartos^*^**

*** Correspondence:** John Tzartos [jtzartos@gmail.com](mailto:jtzartos@gmail.com) or [jtzartos@med.uoa.gr](mailto:jtzartos@med.uoa.gr)

# Supplementary Figures

**
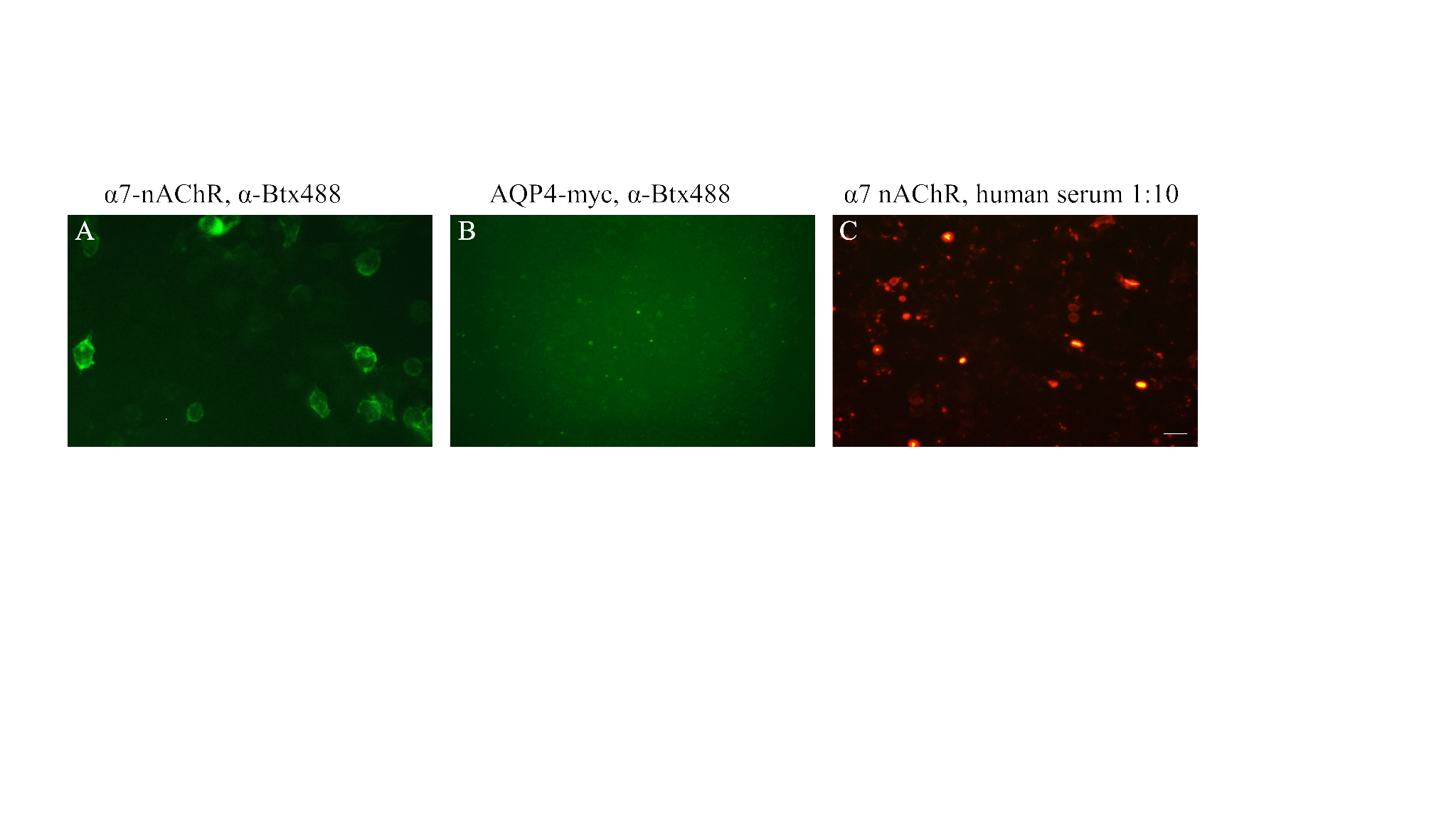
**

**Supplementary Figure 1. α7-AChR Cell-based assay with subunit-specific antagonist.**  HEK293 cells expressing α7-nAChR **(A)** or AQP4 control **(B)**, were co-transfected with NACHO and RIC3 chaperone and cultured in the presence of 1 mM nicotine. Cells expressing α7-nAChR **(A)** or AQP4 control **(B)**, were incubated with fluorescent α-bungarotoxin (specific antagonist for α7-nAChR) **(A-B).**

**Confirmation of serum antibody binding to α4-nAChR by FACS**

To confirm the CBA results assessed by fluorescence microscopy, all positive samples and 10 healthy sera were tested by FACS (representative plots are shown in Suppl. Figure 2). Each sample analysis was performed in duplicate. For analysis, the optimal data acquisition gate was established, and the α4-nAChR-Ab titers were analyzed based on the ratio of mean fluorescence intensity (rMFI) between α4β2-nAChRs-expressing cells versus non-transfected cells. To establish the cut-off value, a threshold was obtained from a cohort of healthy controls, determined by the mean of MFI plus four standard deviations of all the negative samples. The mean rMFI in three positive samples was found to be 3,43 and the proper dilution was determined to be 1:100. The cut off value of healthy serum samples was 1,44.

**
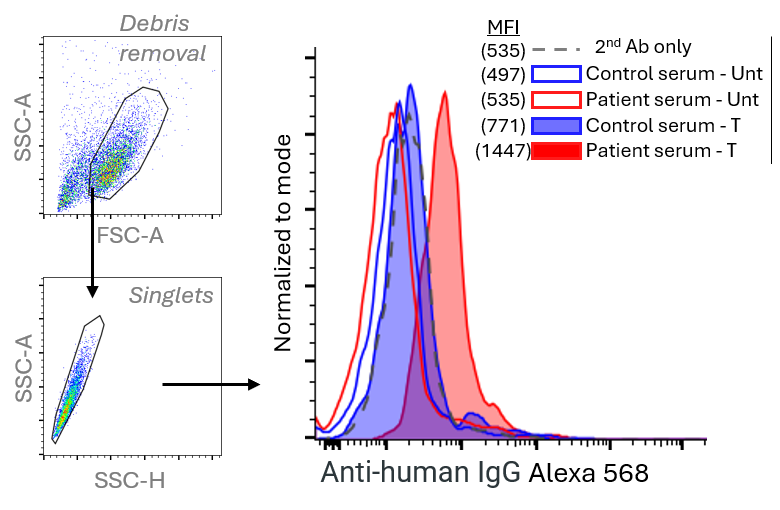
**

**Supplementary Figure 2.** **Confirmation of the presence of** **α4-nAChR-Abs by FACS**. FACS gating and representative examples of α4-nAChR-Ab detection in two patients and two controls. Serum binding to α4β2-nAChR transfected cells was compared to their respective binding to un-transfected cells. The binding of the secondary antibody is also shown. FACS= fluorescence-activated cell sorting;
